# Wernicke’s Encephalopathy Presenting With Confusion in a Patient With Schizophrenia

**DOI:** 10.7759/cureus.32320

**Published:** 2022-12-08

**Authors:** Yui Seo, Mark Reed, Jason S Chang, Forshing Lui

**Affiliations:** 1 Medicine, California Northstate University College of Medicine, Elk Grove, USA; 2 Internal Medicine, California Northstate University College of Medicine, Elk Grove, USA; 3 Neurology, Kaiser Permanente South Sacramento Medical Center, Sacramento, USA; 4 Clinical Sciences, California Northstate University College of Medicine, Elk Grove, USA

**Keywords:** schizophrenia, mental illness, encephalopathy, thiamine deficiency, wernicke’s encephalopathy (we)

## Abstract

Wernicke’s encephalopathy (WE) is a neuropsychiatric condition caused by thiamine deficiency often associated with alcoholism. Other less common causes include prolonged gastroenterology problems or dietary insufficiencies associated with hyperemesis gravidarum, bariatric surgery, and eating disorders. Prolonged WE without proper treatment can lead to the chronic and irreversible condition, Wernicke-Korsakoff syndrome. Despite being known for its classic triad of clinical symptoms (nystagmus/ophthalmoplegia, gait ataxia, and confusion), WE patients more commonly present with non-specific symptoms of altered mental status. Obscure clinical presentations often led to delays in the appropriate of patients with WE. We are presenting a case of WE that is unusual because the underlying cause is schizophrenia and the lack of alcohol use. For a punctual diagnosis, a high index of suspicion is essential to prevent further exacerbation of neuronal death seen in WE. IV thiamine should be administered to any patient with acute encephalopathy or altered mental status, given its low cost and lack of side effects.

## Introduction

Wernicke’s encephalopathy (WE) is a neuropsychiatric condition induced by a deficiency in vitamin B1 (thiamine) - a vital coenzyme in many neurologic biochemical pathways. Depletion of thiamine levels can result in the impairment of thiamine-dependent enzymes such as pyruvate dehydrogenase and transketolase, commonly creating lesions in the thalami, mammillary bodies, and mesencephalon. These selective lesions may lead to the triad of WE symptoms: nystagmus/ophthalmoplegia, gait ataxia, and confusion [1].

While WE carries a significant mortality and morbidity rate, its symptoms can often be quickly reversed through the administration of thiamine. As a result, it has become common practice to give unconscious or heavily inebriated patients thiamine doses before glucose solutions to prevent the precipitation of WE [2].

Thiamine deficiency in WE has been most commonly associated with chronic alcohol abuse in the literature. However, there have been numerous cases of the syndrome unrelated to alcohol drinking. There are reported cases of Wernicke’s encephalopathy following bariatric surgery, GI disorders, excessive vomiting during pregnancy (hyperemesis gravidarum), cancers, systemic diseases, eating disorders, or dietary malnutrition [3].

While WE is known for its traditional triad of symptoms, its actual presentation in clinical practice may be obscured with a plethora of non-specific symptoms, especially common with acute altered mental status or delirium. We are presenting an unusual case of WE in a patient with schizophrenia who presented to the emergency department with altered mental status. Patients with acute altered mental status or delirium will not cooperate well during clinical bedside examination, thus making the signs of ophthalmoplegia and gait ataxia difficult to elicit unless the clinician specifically looks for these signs with a high index of suspicion of WE.

## Case presentation

The patient is a 37-year-old man who was born in Afghanistan. He immigrated to the US with his family at around 10. He graduated from college and joined the US army for two years. He could not hold a steady job subsequently. About 4-5 years ago, he was seen by a psychiatrist due to hallucinations, and a diagnosis of schizophrenia was made. He was placed on aripiprazole. His medication compliance was unknown, yet his psychiatric symptoms have been quite well controlled. He has been unemployed since. The patient has no history of recreational drug use.

In the last three weeks, he has been staying in his room. It was unclear if he was eating or drinking during this period. He was seen going in and out of the bathroom. Over the last week, he was not seen walking, and he was not talking well and appeared confused. His father then called the ambulance, and he was sent to the emergency department and admitted to the hospital.

On admission, the patient was clinically dehydrated with leukocytosis that resolved with IV hydration. His vitals were normal and stable. His BMI was 16.21 kg/m^2^. He had no fever or any other signs of sepsis. He was somnolent and delirious. Nystagmus was noted, and he was unable to walk. The clinical diagnosis was acute encephalopathy, probably secondary to metabolic causes with dehydration. An infection has to be ruled out. The patient’s neurological status did not improve over the next two days with IV hydration. A neurological consult was called.

The patient was seen by the neurologist, who confirmed the findings of delirium with impaired orientation and short-term recall with confabulation. He was perseverative and echolalic. In addition, the patient was noted to have abduction palsy of his right eye and direction-changing horizontal and vertical nystagmus. His strength was grossly intact, yet he was ataxic with total areflexia. He refused to stand and walk.

**Figure 1:**
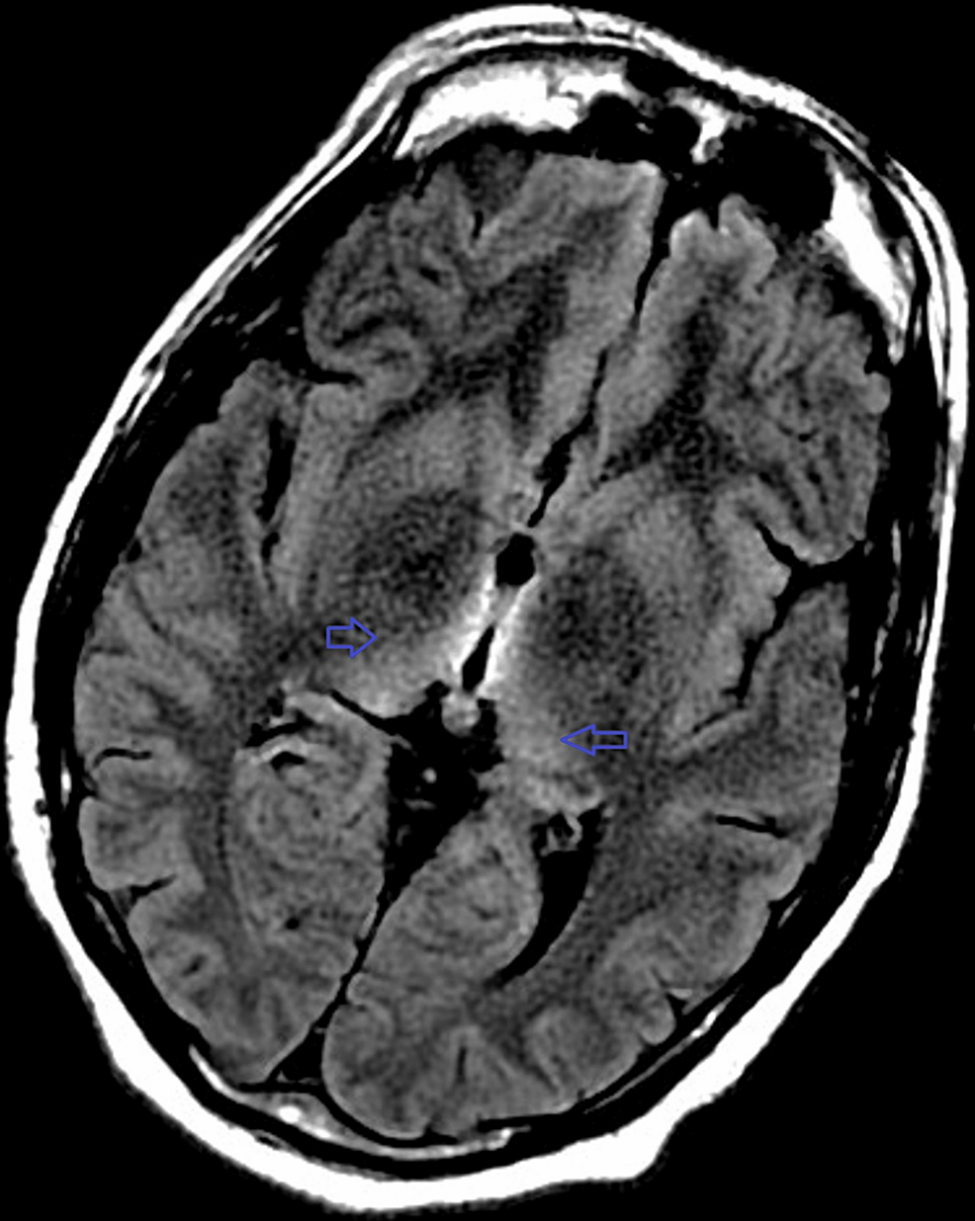
Typical MRI findings of Wernicke's encephalopathy. Bilateral pulvinar signal hyperintensity - hockey stick sign in Wernicke's encephalopathy (arrows).

The neurologist recommended a brain MRI and IV thiamine therapy. The MRI with T2 FLAIR sequence showed classic lesions of WE in the hypothalamus, medial thalamus, pulvinar, and periaqueductal gray matter. The pulvinar showed the characteristic hockey stick sign, as illustrated in Figure 1.

Over the next two days in the hospital, the patient was given thiamine IV 100 mg a day after the initial dose of 100 mg after he was evaluated by the consultant neurologist. The patient improved rapidly with the recovery of his abducens palsy and better mentation. At this time, the patient’s father was reached, and more history was obtained. The patient has apparently been concerned with his body weight since his early 30s. He was lean and athletic when he was younger. After his diagnosis of psychosis and medication treatment, his weight increased to 260 lbs, a gain of 100 lbs. He became self-conscious about his weight, and he lost about 60 lbs in six months of dieting. On admission, his lab results, including blood glucose, electrolytes, complete blood picture including white blood counts, renal function, liver function, total protein, albumin level, total cholesterol, and vitamin B12 levels, are all normal.

## Discussion

WE is caused by a deficiency in thiamine and is commonly associated with alcohol abuse and, less commonly, with dietary insufficiency from other causes. While WE is known for its classic triad (nystagmus/ophthalmoplegia, gait ataxia, and confusion), in reality, these symptoms are only present in approximately 16%-38% of WE patients [1]. Due to its plethora of non-specific symptoms, such as stupor, hypotension, papilledema, hearing loss, hallucinations, epileptic seizures, and behavioral disturbances, it is often difficult to diagnose WE in clinical settings [3]. In addition, various differential diagnoses (Creutzfeldt-Jakob disease, multiple sclerosis, infective encephalopathy, etc.) must be ruled out when weighing risk factors. Therefore, WE from thiamine deficiency must be seriously considered in patients associated with chronic alcoholism, nutrient-poor diets, or bariatric surgery [4].

The commonest recognized psychiatric cause of WE is anorexia nervosa (AN), an eating disorder defined by the DSM-5 as restriction of dietary intake leading to significantly low weight, intense fear of gaining weight, and disturbance in the way one’s own body is experienced [5]. AN affects women more than men by roughly a factor of 10, with a lifetime prevalence in women of up to 4% [6]. Common comorbidities of AN include other psychiatric conditions such as depression, social withdrawal, irritability, insomnia, OCD, and low libido [5].

Recent literature has explored the link between AN and schizophrenia, another DSM-5 psychiatric disorder. Schizophrenia is a psychotic disorder with a lifetime prevalence of about 0.3%-0.7% [5]. The correlation between AN (and, more broadly, a range of eating disorders) and schizophrenia has been demonstrated, primarily in case reports, but the relationship between the two conditions is not well understood [6,7]. Despite the higher prevalence of AN in women when compared to men, comorbidity of schizophrenic symptoms in AN patients is 3.6 times more common in men than in women and may have a prevalence of up to 35% [8]. AN can precede psychosis, arise simultaneously, or present in the residual phase of schizophrenia. Sometimes, AN can arise as a symptom of the pre-psychotic phase of schizophrenia. Other times, the two conditions can have simultaneous symptoms, which makes diagnosis difficult.

Our patient has a history of vivid hallucinations resulting in a diagnosis of schizophrenia. He was treated with a second-generation antipsychotic aripiprazole which has a known side effect of metabolic syndrome and weight gain. He did gain 100 lbs of weight over three years. He tried to lose weight by dietary restrictions resulting in a weight loss of 60 lbs in 5-6 months. This eating pattern culminated in a week of not eating or drinking, resulting in his final clinical problems of “encephalopathy” with dehydration and hospitalization.

WE, in isolation, is a difficult disorder to diagnose, given its non-specific and varied clinical presentation [3]. The overlap of symptoms between WE, eating disorder and schizophrenia further complicated the diagnosis in this patient. For example, hallucinations and visual disturbances are symptoms shared between WE and schizophrenia [3,5]. WE can also cause a lack of appetite, the primary symptom of AN [5,9]. Schizophrenia has also been shown to have an increased risk of undernutrition, with a 21% prevalence [10]. Due to these overlapping symptoms, many patients with WE comorbid with psychiatric disorders remain undiagnosed, leading to worsening and often permanent complications of their symptoms, including the development of Wernicke-Korsakoff Syndrome (WKS) [9].

Literature has shown differences in the prevalence of symptoms in WE patients depending on comorbidities. While the complete classic triad of WE (oculomotor abnormality, gait ataxia, and mental confusion) only arises in 16%-38% of isolated WE cases, when comorbid with schizophrenia or AN, that number rises to approximately 80% (12/15) and 66% (8/12), respectively [1,9,11]. Physicians should be aware that the presence of the complete classic triad could be a useful tool in identifying comorbid psychiatric conditions and WE.

As recent literature has explicated that schizophrenic patients are predisposed to poor eating habits or eating disorders - resulting in malnutrition (in particular, thiamine deficiency) - it is recommended to screen for thiamine levels and to inquire about eating habits regularly during check-ups. If nutritional deficiency or the presence of an eating disorder is suspected, prophylactic thiamine supplementation may prevent the precipitation or exacerbation of thiamine deficiency-induced WE [9,12].

Diagnosis of WE by routine clinical examination is missed in up to 75%-80% of actual cases [13]. Thus, diagnostic yield and sensitivity can improve significantly with brain MRI. The characteristic abnormalities are symmetrical, T2/FLAIR hyperintense lesions in the mammillary bodies, periaqueductal area, thalami, and tectal plate regions [1]. The periaqueductal, hyperintense lesions are often referred to as the “hockey-stick sign” due to their shape. These areas are particularly affected because their osmotic gradients are considered to be closely related to thiamine levels. Other less commonly involved areas of the brain include the brainstem, cerebellum, corpus callosum, fornices, caudate nucleus, and frontal-parietal cortex [14]. The selective lesions present in WE are formed by the lack of sufficient thiamine-dependent, cerebral glucose metabolism - in particular, impaired pyruvate dehydrogenase complex (PDHC), alpha-ketoglutarate dehydrogenase (α-KGDH), and transketolase activity - resulting in depleting ATP levels and increased free radicals [15]. Consequential lactate build-up and cerebral edema induce injury of myelin sheaths, impair axonal conduction, and disturb the blood-brain barrier, causing neuron death - leading to the typical triad of nystagmus, gait ataxia, and confusion often observed in WE patients [16].

Without prompt treatment of this condition, WE can progress from an acute, reversible affliction to a chronic, more permanent WKS. Even with the administration of thiamine, WKS may develop, characterized by a series of personality changes, learning deficits, confabulation, and/or anterograde and retrograde amnesia (or related memory deficits). Left untreated, WE has a mortality rate of approximately 17% of patients. Thus, it is critical to promptly identify the clinical presentation of WE to minimize cerebral damage and to recognize high-risk cases, including patients with psychiatric illness, to administer prophylactic thiamine.

## Conclusions

WE is a relatively common problem. The diagnosis is not difficult in an alcoholic with the classical triad of confusion, ophthalmoplegia, and ataxia. However, the diagnosis is often missed in a non-alcoholic who presents only with mental status changes, resulting in delayed diagnosis, treatment, and worsened prognosis. Our patient presented with an important confounding factor of a psychotic disorder of schizophrenia which is very seldom recognized as an important cause of thiamine deficiency and WE.

Our patient also demonstrated the apparent difficulties associated with an early and correct diagnosis. He is not an alcoholic with no apparent history of dietary deficiency on admission. His presentation of dehydration and history of functional psychosis further complicates the clinical diagnosis and judgment. The diagnosis was finally made by the consulting neurologist, aided by typical MRI findings. Therefore, it is recommended that physicians remain vigilant about such an association with mental health disorders, especially schizophrenia or eating disorder. It is also advisable for clinicians to have an adequate and regular screening of patients at risk for developing diet-associated WE.
